# Health Insurance and Colorectal Cancer Survival in Khon Kaen, Thailand

**DOI:** 10.31557/APJCP.2019.20.6.1797

**Published:** 2019

**Authors:** Surachai Phimha, Supannee Promthet, Krittika Suwanrungruang, Jarin Chindaprasirt, Prachak Bouphan, Chalongpon Santong, Patravoot Vatanasapt

**Affiliations:** 1 *Doctor of Philosophy Program in Epidemiology and Biostatistics, *; 5 *Department of Public Health Administration Health Promotion Nutrition, Faculty of Public Health, *; 2 *ASEAN Cancer Epidemiology and Prevention Research Group, *; 3 *Cancer Unit, Srinagarind Hospital, *; 6 *Department of Otorhinolaryngology, Faculty of Medicine, *; 4 *Division of Oncology, Department of Internal Medicine, Faculty of Medicine, Khon Kaen University, Khon Kaen, Thailand. *

**Keywords:** Health insurance, colorectal cancer, relative survival

## Abstract

**Background:**

Evidence from healthcare studies demonstrates that patients’ health insurance affects service accessibility and the outcome of treatment. However, assessment on how colorectal cancer survival relates to health insurance is limited.

**Objective:**

The study examined the association between health insurance and colorectal cancer survival in Khon Kaen, Thailand.

**Methods:**

The retrospective cohort study was conducted with 1,931 colorectal cancer patients from Khon Kaen cancer registry between January 1, 2003 and December 31, 2012, and was followed-up until December 31, 2015. Relative survival was used to estimate the survival rate. Cox proportional hazard regression was used to estimate the relationship between health insurance and colorectal cancer survival, represented with the hazard ratio.

**Result:**

Most of the participants were males, and the median age was 62 years. The median survival time was 2.25 years (95% CI: 2.00-2.51). The five-year observed survival rate and relative survival rate were 36.87 (95% CI: 34.66-39.08) and, 42.28 (95% CI: 39.75-44.81), respectively. The factors that showed significant associations with poorer survival after adjustment for gender and age were non-surgical treatments (HRadj=1.88;95%CI=1.45-2.45), advanced stage (III+IV) (HRadj=2.50; 95%CI=2.00-3.12), histological grading in poorly differentiated (HRadj=1.84; 95%CI=1.32-2.56), and Universal Coverage Scheme (HRadj=1.37;95%CI=1.09-1.72).

**Conclusion:**

The survival of colorectal cancer patients in the Universal Coverage Scheme was likely to be poorer than in the Civil Servant Medical Benefit Scheme. This indicates an urgent need for a national program for colorectal cancer screening in the general population and access to health insurance.

## Introduction

Colorectal cancer (CRC) kills more than 600,000 people a year worldwide, and is one of the most common types of cancer (Wu et al., 2016). The global burden of CRC is expected to increase by 60%, with more than 2.2 million new cases and 1.1 million deaths by 2030 (Arnold et al., 2017). An increasing trend of CRC incidence is found in Asia, where nearly 45% of cases worldwide occur (Chiu et al., 2015). 

In Thailand, CRC is the third most common type of cancer in males (The age-standardised incidence rate (ASR) is 15.2 per 100,000 population) and the fifth most common in females (ASR = 10.1 per 100,000 population) [International Agency for Research on Cancer (IARC), 2017]. 

The treatment choices and survival of CRC patients were found to differ according to the coverage of health insurance (Roetzheim et al., 2000). A previous study in Kentucky showed that CRC patients with Medicare health insurance had a 32% higher risk of death than patients who were privately insured; Medicaid welfare insurance patients had a 56% higher risk; patients with unknown health insurance had a 66% greater risk (McDavid et al., 2003). 

For half a century, the Thai government has attempted to expand health-care coverage for the Thai population. In 1975, low-income populations were exempted from hospital charges (the “low-income scheme”). In 1992, the program was expanded to other groups such as children younger than 12 and the elderly, and a publicly subsidised health insurance scheme (the Voluntary Health Card) was created (Tangcharoensathien et al., 2004). The Civil Servant Medical Benefit Scheme (CSMBS) was created in 1980 to support healthcare services for government employees, dependents and their families. In 1990, a Social Security Scheme (SSS) was launched to cover employees who worked in private sectors. In 2001, about 30% of the Thai population lacked health insurance. As a result, the government launched a Universal Coverage Scheme (UCS) in October 2001. This program combined the Low-Income Scheme and the Voluntary Health Card into a single scheme. This reform meant that all the Thai population had healthcare coverage either by CSMBS, SSS, or UCS (Health Insurance System Research Office, 2012; Thailand Development Research Institute, 2013).

The five-year survival rate of CRC patients in Khon Kaen, Thailand was 26.50% in 2016 (Siewchaisakul et al., 2016). This was lower than in 2010, when the rate was 38.6%, (Laohavinij et al., 2010). This study aims to determine the association between health insurance schemes and colorectal cancer survival rates in Khon Kaen province, Thailand.

## Materials and Methods

This retrospective cohort study assessed all new cases of CRC registered in the population-based cancer registry of Khon Kaen province, according to the International Classification of Diseases for Oncology (ICD-O, third edition) from C18.0 for cecum to C20.9 for rectum. The data were retrieved for those diagnosed between January 1, 2003 and December 31, 2012. Death-certificate only cases and those with multiple primaries were excluded. The factors of interest were health-insurance including the Civil Servant Medical Benefit Scheme (CSMBS), the Social Security Scheme (SSS), and the Universal Coverage Scheme (UCS).

*Follow–up*

The last vital status of participants was updated by linkage with the National Health Security Office (NHSO), Thailand. The medical data was obtained from individual medical records and the time of observation was until death or the end of the study period in December 31, 2015.

*Statistical analysis*

Descriptive statistics were analysed and presented the demographic characteristics with numbers and percentages for categorical data. The continuous data were analysed and presented by mean, standard deviation (SD) and interquartile range (IQR). Observed survival rates were calculated by the actuarial life table. Relative survival rates were calculated by dividing the observed survival rates by the expected survival rates estimated by the generation life tables for Thailand (Spika et al., 2018).

Cox proportional hazard regression was used to estimate the relationship between factors and outcomes represented with a hazard ratio (HR) with a confidence interval at 95%, and the p-value was described by a partial likelihood ratio test at a significant level of 0.05. The initial multivariable model from a univariate analysis considered factors with a p-value < 0.25, and important factors from review literature. The data were analysed by backward elimination to find the final model. The data analysis was adjusted by factors including gender and age. The proportional hazard assumption test and goodness of fit test were used to test for the fit model.

The data were assessed using the STATA program version 15.0 (copyright Faculty of Public Health, Khon Kaen University.

*Ethical considerations*

This study was approved by Khon Kaen University Ethics Committee for Human Research based on the Declaration of Helsinki and the ICH Good Clinical Practice Guidelines. The reference number is HE 611218.

**Figure 1:**
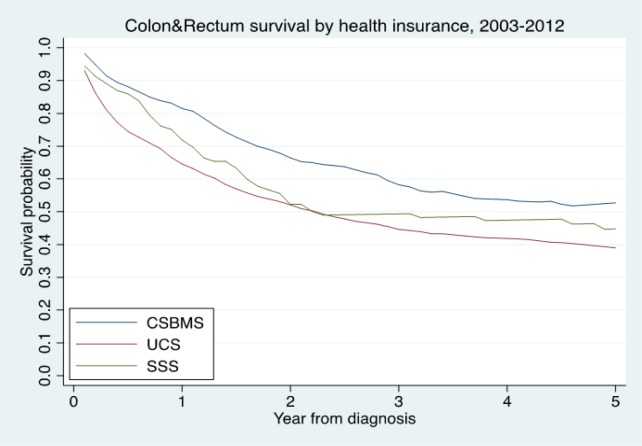
The Relative Survival Times by Health Insurance

**Table 1:** Demographic Data of Colorectal Cancer Patients

Variables	Number (1,931)	%
Gender		
Male	1,021	52.87
Female	910	47.13
Age(years)		
< 50 years	372	19.26
50-59 years	448	23.2
> 60 years	1,111	57.54
Mean (S.D.)	61.45(13.29)	
Median (Q1:Q3)	62 (52:71)	
Tumour sites		
Left-sided colon	259	13.41
Right-sided colon	508	26.31
NOS Colon	506	26.2
Rectum, NOS	658	34.08
Tumour sites group		
Colon	1,147	59.4
Rectum	784	40.6
Stage of disease		
Stage I	45	2.33
Stage II	282	14.6
Stage III	355	18.39
Stage IV	478	24.75
Unknown stage	771	39.93
Histological type		
Adenocarcinoma	1,452	75.19
Non-adenocarcinoma	479	24.81
Histological grading (1,197)		
Well differentiated	842	70.34
Moderately differentiated	278	23.23
Poorly differentiated	77	6.43
Treatment		
Surgery	1,300	67.32
Non-surgery	631	32.68
Health insurance (1,883)		
CSMBS	386	20.5
UCS	1,407	74.72
SSS	90	4.78

CSMBS, civil servant medical benefit scheme; UCS, universal coverage scheme; SSS, social security scheme

**Table 2:** Survival Rate of Colorectal Cancer Patients

Survival time	Male		Female		Both genders	
	OS (95% CI)	RS (95% CI)	OS (95% CI)	RS(95% CI)	OS (95% CI)	RS (95% CI)
1 year	64.04 (61.00-66.91)	66.19 (63.06-69.13)	69.08 (65.95-71.98)	70.93 (67.75-73.89)	66.42 (64.26-68.49)	68.43 (66.22-70.55)
3 years	42.6 (39.54-45.63)	46.65 (43.30-49.96)	46.1 (42.82-49.32)	49.22 (45.71-52.65)	44.25 (42.02-46.47)	47.86 (45.44-50.25)
5 years	34.8 (31.78-37.82)	40.68 (37.17-44.21)	39.16 (35.91-42.40)	44.03 (40.38-47.65)	36.87 (34.66-39.08)	42.28 (39.75-44.81)

CI, confidence interval; OS, overall survival; RS, relative survival.

**Table 3:** Factors Related to Mortality of Colorectal Cancer (Multivariate Analysis)*

Variable	Number (%)	Median time (Year) 95% CI	Crude HR (95% CI)	Adj. HR (95% CI)	p-value**
Health insurance					<0.0001
CSMBS	386 (20.50)	3.29 (2.84-4.40)	1	1	
UCS	1,407 (74.72)	1.98 (1.69-2.25)	1.36 (1.18-1.57)	1.37 (1.09-1.72)	
SSS	90 (4.78)	2.10 (1.57-5.69)	1.04 (0.78-1.40)	0.78 (0.48-1.29)	
Combined Stage					<0.0001
Stage I+II	327 (28.19)	11.43 (7.59-NA)	1	1	
Stage III+IV	833 (71.81)	1.56 (1.37-1.73)	3.05 (2.51-3.72)	2.50 (2.00-3.12)	
Treatment					<0.0001
Surgery	1,300 (67.32)	3.42 (2.90-4.17)	1	1	
Non-Surgery	631 (32.68)	0.79 (0.62-1.00)	2.09 (1.86-2.34)	1.88 (1.45-2.45)	
Histological grading					<0.0001
Well	842 (70.34)	3.86 (3.10-4.67)	1	1	
Moderately	278 (23.23)	2.55 (2.01-3.22)	1.19 (1.00-1.42)	1.24 (1.00-1.52)	
Poorly	77 (6.43)	0.98 (0.45-1.49)	2.33 (1.79-3.04)	1.84 (1.32-2.56)	
Tumour site group					-
Colon	1,147 (59.40)	2.50 (2.07-2.92)	1	-	
Rectum	784 (40.60)	2.01 (1.71-2.35)	1.17 (1.05-1.31)	-	
Histological type					-
Adenocarcinoma	1,452 (75.19)	2.89 (2.53-3.22)	1	-	
Non-adenocarcinoma	479 (24.81)	0.86 (0.59-1.16)	1.72 (1.52-1.95)	-	

*, adjusting for gender and age; **, p-value from partial likelihood ratio test

**Figure 2:**
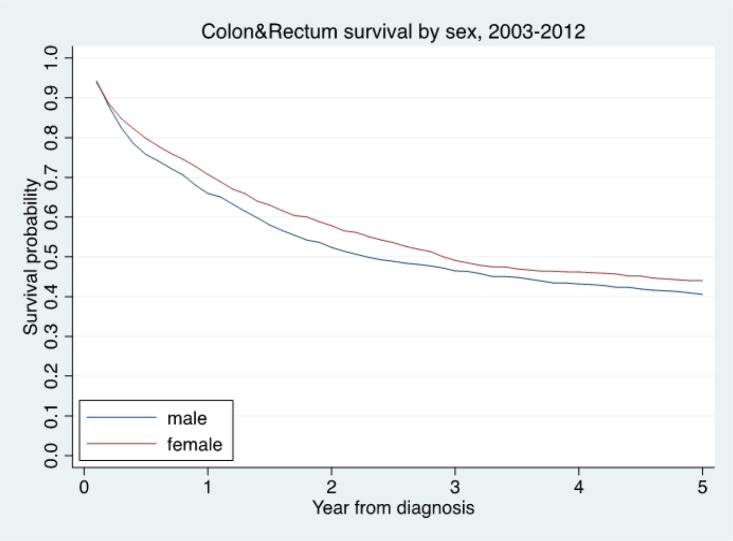
The Relative Survival Times by Sex

**Figure 3:**
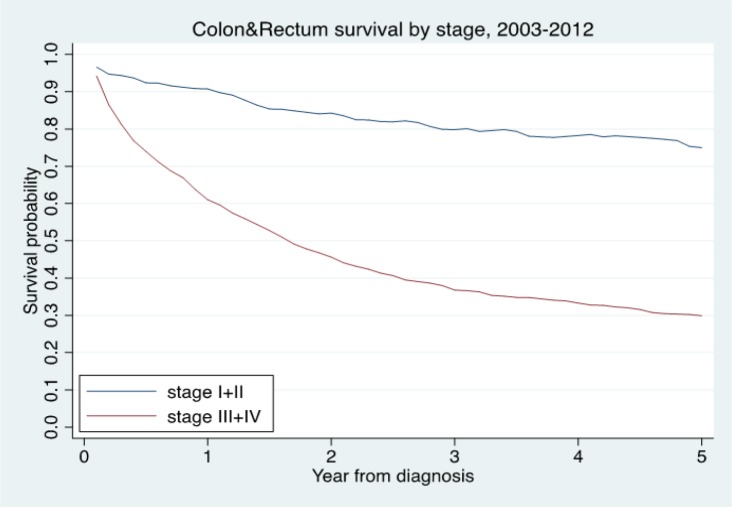
The Relative Survival Times by Stage Group

## Results

*Patient characteristics*

Most cases were males; the median (Q1:Q3) age was 62 (52:71) years. The highest percentage of common tumour site, stage, and histological types were the colon (59.40%), stage IV (24.75%) and adenocarcinoma (75.19%), respectively. The majority (67.32%) underwent surgery for treatment. The UCS health insurance program was higher than other programs (Table 1).

*Survival rate*

The overall follow-up person-time was 6,398.70 person-years, and the overall mortality rate was 19.64 per 100 person-years (95%, CI=18.59-20.76). The median survival time was 2.25 years (95%, CI=2.00-2.51). The median time for early stage (I+II) was 11.43 (95%, CI=7.59-NA) years and advanced stage (III+IV) was 1.56 (95%, CI=1.37-1.73) years. The median survival times for patients with CSMBS, UCS and SSS health insurance were 3.29 (95%, CI=2.84-4.40), 1.98 (95%, CI=1.69-2.25) and 2.10 (95%, CI=1.57-5.69) years, respectively (Table 3). The one, three and five-year observed survival rate and relative survival rate were 36.87 (95%, CI=34.66-39.08) and 42.28 (95%, CI= 39.75-44.81) (Table 2). The figure shows the overall relative survival rate (Figures 1-3).

*Factors associated with mortality*

We found that the UCS program was significantly associated with poorer survival compared with CSMBS (HRadj =1.37; 95%, CI=1.09-1.72). The other factors significantly associated with poorer survival after adjusting for gender and age included non-surgery (HRadj= 1.88; 95%, CI=1.45-2.45), advanced stage (III+IV) (HRadj= 2.50; 95%, CI=2.00-3.12), and poorly differentiated histological grading (HRadj=1.84; 95%, CI=1.32-2.56) (Table 3).

## Discussion

*This is the first study determining the survival benefits of health insurance on CRC in Thailand.*

Our main finding indicates that UCS has the worst prognosis compared with other schemes. This is supported by a United States study which showed that Medicaid health insurance is associated with a higher risk of mortality than private health insurance (Roetzheim et al., 2000; McDavid et al., 2003; Robbins et al., 2009; Robbins et al., 2010; Niu et al., 2013; Tawk et al., 2016). It was hypothesised that the hospital charges of CRC treatment were higher for those who were on CSMBS than UCS and SSS (Chindaprasirt et al., 2012). People’s health-seeking behaviour did not change much after the UCS insurance started because choices were still limited. Villagers tended to buy medicine at a nearby dispensary rather than to go to a hospital that was far away. (NaRanong and NaRanong, 2006). The people who got UCS had to obtain healthcare services at the contracted hospital under the district health system (Health Insurance System Research Office, 2012), while CSMBS member could obtain healthcare services from any public hospital nationwide (Thailand Development Research Institute, 2013). Moreover, the increasing distance between home and hospital was found to be a barrier to accessing healthcare (Nemet and Bailey, 2000; Jordan et al., 2004). 

In 2013 oxaliplatin, a cytotoxic drug, was approved on the National List of Essential Medicines to treat stage III CRC in all schemes (National Drug System Development committee, 2013). As a result, in the future CRC prognosis of UCS is likely to improve. In addition, treatment with non-surgery was significantly associated with an increased risk of CRC death, and this corresponds to the previous study in the United States (Roetzheim et al., 2000; Hofmann et al., 2010). The poorly differentiated had a significant association with increased risk of CRC death. This is also in line with the study in Thailand (Laohavinij et al., 2010; Siewchaisakul et al., 2016) and in the United States (Hofmann et al., 2010; Robbins et al., 2010). 

Unsurprisingly, we found tumour advanced stage (III+IV) had a significant association with an increased risk of CRC death. This finding was similar to the previous study, which reported that the last stage had a higher risk of death than the early stage (Koo et al., 2008; Laohavinij et al., 2010; Siewchaisakul et al., 2016; Magaji et al., 2017).

The five-year relative survival rate from this finding was 42.45% (39.88-45.00). This rate is consistent with previous studies reported in Thailand. From 1993 to 1997, Sankaranarayanan et al., (2011) reported a five-year relative survival rate in colon and rectum patients of 43.3% and 42.8 %, respectively. These figures were close to the colon relative survival rates in China and the Philippines (five-year RS; 1990-2001; 44% and 40%, respectively). However, the five-year relative survival from this study is lower than in South Korea (five-year RS 1990-2001, Singapore, 60%, 52%, Sankaranarayanan et al., 2010), Finland, Sweden, and England (five-year RS 2000-2002 59.1%, 60.3%, 52.7%, respectively, Brenner et al., 2012). It is also less than in Switzerland (five-year RS 2005-2009 61%, Bordoni et al., 2012), and the United States (five-year RS 2003-2009 64.9%, Siegel et al., 2014).

The prognosis of CRC patients in this study was found to be poorer because the majority (71.81%) were diagnosed in advanced stages, while the proportion of advanced stage CRC in developed countries was 0.45 in the United States (Hofmann et al., 2010) and 0.48 in Japan (Tamakoshi et al., 2017). In Thailand, a policy for national CRC screening was launched in 2018, but this has not yet been implemented nationwide. In European Union (EU) countries 19 of 27 have a screening program (Zavoral et al., 2009). CRC screening in the United States has been in place since the 1960s or earlier (Doubeni, 2014). In Asia a CRC screening program operates in Japan (1992), Korea (2004), and Singapore (2009) (Chiu et al., 2017). In Taiwan a nationwide CRC screening program was launched in 2004. This led to a reduction of CRC mortality in the first decade of the program (Wang et al., 2017).

In conclusion, this study found that the survival rate of CRC patients was not improved by having expanded healthcare access in Thailand, and was still lower than in other developed countries in Asia and Europe. Moreover, the survival rate of CRC patients in the UCS is likely to be poorer than in the CSMBS. This indicates an urgent need for a national program for CRC screening in the general population.
